# Coccidioidomycosis Masquerading as Eosinophilic Ascites

**DOI:** 10.1155/2015/891910

**Published:** 2015-07-21

**Authors:** Kourosh Alavi, Pradeep R. Atla, Tahmina Haq, Muhammad Y. Sheikh

**Affiliations:** Division of Gastroenterology and Hepatology, University of California San Francisco, 1st Floor, Endoscopy Suite, 2823 Fresno Street, Fresno, CA 93721, USA

## Abstract

Endemic to the southwestern parts of the United States, coccidioidomycosis, also known as “Valley Fever,” is a common fungal infection that primarily affects the lungs in both acute and chronic forms. Disseminated coccidioidomycosis is the most severe but very uncommon and usually occurs in immunocompromised individuals. It can affect the central nervous system, bones, joints, skin, and, very rarely, the abdomen. This is the first case report of a patient with coccidioidal dissemination to the peritoneum presenting as eosinophilic ascites (EA). A 27-year-old male presented with acute abdominal pain and distention from ascites. He had eosinophilia of 11.1% with negative testing for stool studies, HIV, and tuberculosis infection. Ascitic fluid exam was remarkable for low serum-ascites albumin gradient (SAAG), PMN count >250/mm^3^, and eosinophils of 62%. Abdominal imaging showed thickened small bowel and endoscopic testing negative for gastric and small bowel biopsies. He was treated empirically for spontaneous bacterial peritonitis, but no definitive diagnosis could be made until coccidioidal serology returned positive. We noted complete resolution of symptoms with oral fluconazole during outpatient follow-up. Disseminated coccidioidomycosis can present in an atypical fashion and may manifest as peritonitis with low SAAG EA. The finding of EA in an endemic area should raise the suspicion of coccidioidal dissemination.

## 1. Introduction

Eosinophilic ascites (EA) is generally a rare finding in clinical practice. When present, it is most commonly associated with migrant parasitic infections, neoplasms, peritoneal dialysis, and eosinophilic gastroenteritis (EGE) [1, 2]. Previous cases described in the literature indicate that intra-abdominal coccidioidomycosis (IAC) can clinically present in a variety of ways ranging from an incidentally found asymptomatic indolent form to a full-blown acute abdominal process and may even mimic an occult malignancy [3–5]. We propose in this report that an even more rare cause of EA is IAC with peritoneal involvement. To the best of our knowledge, this is the first report of its kind.

## 2. Case Report

A 27-year-old male immigrant from Mexico (BMI of 25.3 kg/m^2^) with past medical history of constipation presented to the emergency room (ER) with abdominal pain, distention, and difficulty in breathing. His symptoms started 9 days prior to the visit. He had two prior ER visits for abdominal pain that was attributed to constipation but the symptoms persisted even after he had bowel movements. Associated symptoms included fatigue, dry cough for one month, and weight loss. He usually weighs around 130 pounds and reports weight loss of about 20 pounds. He denied fever, chills, night sweats, nausea, bloody stool, diarrhea, headache, or chest pain. He admitted to heavy alcohol use but quit about 4 months ago. There was no history of recent travel, sick contacts, transfusions, allergy, smoking, and illicit drug use. His physical exam was significant for distended abdomen with tense ascites and diffuse tenderness. There were no stigmata of chronic liver disease and no evidence of pedal edema. He weighed 125 pounds on initial presentation; however, his weight loss was apparent after paracentesis with 115 pounds after the removal of 4 liters of ascitic fluid. Abnormal laboratory values and the results for the paracentesis are listed in Tables 1 and 2.

Abdominal imaging with ultrasound and computed tomography (CT) scan showed ascites and thickened small bowel wall without evidence of bowel obstruction, cirrhosis, splenomegaly, and portal or hepatic venous thrombosis. Additionally, atelectasis of the lung bases (especially on the left) was seen on imaging. Transthoracic echocardiogram of the heart was normal.

Differential diagnoses considered were SBP and tuberculous peritonitis. Curiously due to the elevated ascitic fluid eosinophils of 62% we also considered parasitic infections, hypereosinophilic syndrome (HES), and eosinophilic gastroenteritis (EGE) as other possible etiologies. Additionally, because our patient was from an endemic area for coccidioidomycosis, disseminated infection with peritoneal involvement was considered as well and the necessary serological tests were requested on presentation while other etiologies were being evaluated.

We started empiric treatment with antibiotics (Ceftriaxone) for SBP with modest clinical improvement. Disseminated tuberculosis was ruled out following a negative Quantiferon-Gold TB test, absence of typical symptoms, and negative ascitic fluid results. Parasitic infections were also ruled out following a negative stool test for ova and parasite. Moreover, HES was unlikely as there was no evidence of organ failure.

We pursued upper gastrointestinal endoscopy and repeated with push enteroscopy to evaluate for EGE. However, gastric and duodenal biopsies were negative for malignancy, increased eosinophils, and Helicobacter pylori infection. Jejunal biopsies were negative for malignancy, ulcer, abnormal inflammation, or Giardia-like organisms.

Subsequent to these and a host of other negative diagnostic test results (Table 3), the existence of marked EA in our young patient, who had no history of any liver disease, allergy, or drug use, especially in the setting of significant weight loss, became worrisome for an occult malignancy. Therefore, a bone marrow biopsy was added to the plan; however, it was never done since the patient's clinical condition continued to improve on SBP treatment. In retrospect, our concern for malignancy was grounded since there is evidence that extrapulmonary coccidioidomycosis can occasionally mimic symptoms of an occult malignancy with operative findings of an inflamed peritoneum that is studded with nodular lesions [4, 5]. We later added steroids to the treatment as lingering suspicion for EGE still remained. Diagnostic dilemma existed for this patient throughout the visit while the result for coccidioidal serology was pending. Eventually the patient was discharged on steroids with outpatient follow-up in the clinic. Soon after discharge, his serology came positive for IgG and IgM antibodies against* Coccidioides immitis*. His steroids were discontinued and treatment was started with 400 mg of fluconazole PO daily. He showed remarkable improvement in the subsequent clinic follow-up after discharge. His ascites has completely resolved and he has started to gain weight with improved weight of 120 pounds at 3 months after discharge.

## 3. Discussion

*Coccidioides* species are dimorphic soil-dwelling saprophytes that are predominantly found in the southwestern parts of the United States. In dry seasons these organisms remain dormant in the soil, but when the rains come, they grow into long filamentous molds that can break off and give rise to airborne spores. Inhalation of these airborne spores can cause infection [6].

Of all the individuals who become exposed to* Coccidioides immitis*, only about approximately 40% become symptomatic with the symptoms being mainly limited to the lungs. Only less than 1% develop widespread disseminated infection [7]. The main risk factors for developing the disseminated form of the disease include immunosuppression, HIV infection, pregnancy, skin test anergy, sex (male), and being of Filipino or African ancestry [7, 8]. Peripheral eosinophilia has been proposed to be an indicator of disseminated disease [9]. In fact, it has been suggested that, in a patient with coccidioidomycosis, extreme eosinophilia may be the only indicator of disseminated disease [10]. Typically the extrapulmonary symptoms of disseminated disease appear a few months after the primary infection [11]. The CNS, skin, joints, and bones are the most common sites of extrapulmonary dissemination but almost any site may be infected [7, 8, 12]. Involvement of the abdomen, however, is rare and was first reported in 1939 by Ruddock and Hope [13]. The presentation of IAC is nonspecific and generally imitates other infections or malignancy and the diagnosis mainly relies on tissue examination [4, 5, 14].

IAC most commonly involves the peritoneum, but involvement of liver, spleen, small bowel, appendix, and the adnexa of the uterus has also been reported [3, 14, 15]. Peritoneal coccidioidomycosis can clinically present in a variety of ways ranging from an incidentally found asymptomatic indolent disease to a full-blown acute abdominal process with peritoneal irritation and ascites [3]. Abdominal pain, diarrhea, ileus, and various constitutional symptoms, such as low-grade fever, malaise, nausea, and weight loss (or weight gain from the ascites), may also be present [15]. Diagnosis is based on histopathological examination as well as ascitic fluid or peritoneal tissue cultures [3]. Serologic [16] and laparoscopic [17] examinations have also been reported to provide critical information for making the diagnosis; however, to the best of our knowledge, our report is the first to highlight EA as an important clue to the diagnosis of IAC.

Azole antifungal medications (e.g., fluconazole, ketoconazole, and itraconazole) are considered first line agents in the treatment of various forms of pulmonary as well as disseminated coccidioidal infections [18].

## 4. Conclusion

Intra-abdominal coccidioidomycosis is very rare even in the endemic areas and can present with eosinophilic ascites as the only apparent clinical manifestation. Healthcare providers need to be aware of this rare presentation to avoid misdiagnosis and unnecessary testing. Azole antifungal medications such as fluconazole are considered first line agents for treatment.

## Figures and Tables

**Table 1:** Laboratory characteristics.

Parameter	Value	Normal range
Complete blood count		
HGB	13.9 g/dL	13.5–17.5 g/dL
Platelet	457 K/*μ*L	150–450 K/*μ*L
Lymphocytes	13.7%	25–45%
Eosinophils	11.1%	0–6%
Electrolytes		
Bicarbonate	29 mEq/L	22–28 mEq/L
Calcium (ionized)	3.43 mg/dL	4.4–5.4 mg/dL
Liver function		
Albumin	3.2 g/dL	3.5–5.5 g/dL
Globulin	4.0 g/dL	2–3.5 g/dL
Iron		
Ferritin	387 ng/mL	20–250 ng/mL
Serum iron	23 *μ*g/dL	65–177 *μ*g/dL
TIBC	193 *μ*g/dL	250–370 *μ*g/dL
Iron saturation	12%	20–50%
Coagulation profile		
PT	14.7 sec	12–14.7 sec
INR	1.1	0.8–1.1
Miscellaneous		
Lipase	18 U/L	12–53 U/L
CRP	88 mg/L	<1 mg/L
ESR	46 mm	<15 mm/hr
LDH		
Serum IgE	530 IU/mL	0–380 IU/mL
HIV screen (ELISA)	Nonreactive	Nonreactive
Anti-cocci antibody	Positive	Negative
Quantiferon-TB Gold	Negative	Negative
Urinalysis		
Protein	50 mg/dL	Negative
Ketones	4+	Negative
Stool analysis		
Ova and parasite	Negative	Negative
Cultures	Negative	Negative

Only abnormal lab values are listed. HGB: hemoglobin; TIBC: total iron binding capacity; CRP: C-reactive protein; ESR: erythrocyte sedimentation rate; PT: prothrombin time; INR: international normalized ratio; LDH: lactate dehydrogenase; HIV: human immunodeficiency virus; ELISA: enzyme-linked immunosorbent assay.

**Table 2:** Characteristics of ascitic fluid.

Parameter	Value
Appearance	Hazy
Color	Yellow
Volume	1000 mL
White blood cells	4095 mm^3^
Red blood cells	1884 mm^3^
Neutrophils	7%
Lymphocyte	25%
Monocyte	1%
Macrocyte	3%
Eosinophils	62%
Mesothelial cells	2%
Culture	No growth
Gram stain	No organism
Protein	5.2 gm/dL
Albumin	2.2 g/dL
Amylase	11 U/L
Lactate dehydrogenase	118 IU/L
Glucose	47 mg/dL
Cytology	No malignancy

**Table 3:** Autoimmune laboratory testing.

Parameter	Result
Anti-SCL-70 antibody	Negative
Anti-Smith antibody	Negative
Anti-nRNP antibody	Negative
Anti-dsDNA antibody	Negative
Anti-SSA/Ro antibody	Negative
Anti-SSB/La antibody	Negative
Anti-smooth muscle antibody	Negative
Anti-mitochondrial antibody	Negative
Anti-LKM antibody	Negative
C-ANCA antibody	Negative
P-ANCA antibody	Negative

LKM: liver kidney microsomal antibody; ANCA: anti-nuclear cytoplasmic antibody.

## References

[1] Copeland B. H., Aramide O. O., Wehbe S. A., Fizgerald S. M., Krishnaswamy G. (2004). Eosinophilia in a patient with cyclical vomiting: a case report. *Clinical and Molecular Allergy*.

[2] Chen M.-J., Chu C.-H., Lin S.-C., Shih S.-C., Wang T.-E. (2003). Eosinophilic gastroenteritis: clinical experience with 15 patients. *World Journal of Gastroenterology*.

[3] Phillips P., Ford B. (2000). Peritoneal coccidioidomycosis: case report and review. *Clinical Infectious Diseases*.

[4] Ellis M. W., Dooley D. P., Sundborg M. J., Joiner L. L., Kost E. R. (2004). Coccidioidomycosis mimicking ovarian cancer. *Obstetrics and Gynecology*.

[5] Eyer B. A., Qayyum A., Westphalen A. C., Yeh B. M., Joe B. N., Coakley F. V. (2004). Peritoneal coccidioidomycosis: a potential CT mimic of peritoneal malignancy. *Abdominal Imaging*.

[6] Nguyen C., Barker B. M., Hoover S. (2013). Recent advances in our understanding of the environmental, epidemiological, immunological, and clinical dimensions of Coccidioidomycosis. *Clinical Microbiology Reviews*.

[7] Ampel N. M., Wieden M. A., Galgiani J. N. (1989). Coccidioidomycosis: clinical update. *Reviews of Infectious Diseases*.

[8] Chiller T. M., Galgiani J. N., Stevens D. A. (2003). Coccidioidomycosis. *Infectious Disease Clinics of North America*.

[9] Echols R. M., Palmer D. L., Long G. W. (1982). Tissue eosinophilia in human coccidioidomycosis. *Reviews of Infectious Diseases*.

[10] Harley W. B., Blaser M. J. (1994). Disseminated coccidioidomycosis associated with extreme eosinophilia. *Clinical Infectious Diseases*.

[11] Chung C. R., Lee Y. C., Rhee Y. K. (2011). Pulmonary coccidioidomycosis with peritoneal involvement mimicking lung cancer with peritoneal carcinomatosis. *American Journal of Respiratory and Critical Care Medicine*.

[12] Stevens D. A. (1995). Coccidioidomycosis. *The New England Journal of Medicine*.

[13] Ruddock J. C., Hope R. B. (1939). Coccidioidal peritonitis: diagnosis by peritoneoscopy. *The Journal of the American Medical Association*.

[14] Micha J. P., Goldstein B. H., Robinson P. A., Rettenmaier M. A., Brown J. V. (2005). Abdominal/pelvic Coccidioidomycosis. *Gynecologic Oncology*.

[15] Smith G., Hoover S., Sobonya R., Klotz S. A. (2011). Abdominal and pelvic coccidioidomycosis. *The American Journal of the Medical Sciences*.

[16] Adam R. D., Elliott S. P., Taljanovic M. S. (2009). The spectrum and presentation of disseminated coccidioidomycosis. *The American Journal of Medicine*.

[17] Jamidar P. A., Campbell D. R., Fishback J. L., Klotz S. A. (1992). Peritoneal coccidioidomycosis associated with human immunodeficiency virus infection. *Gastroenterology*.

[18] Catanzaro A., Galgiani J. N., Levine B. E. (1995). Fluconazole in the treatment of chronic pulmonary and nonmeningeal disseminated coccidioidomycosis. *The American Journal of Medicine*.

